# In Vitro and In Vivo Imaging-Based Evaluation of Doxorubicin Anticancer Treatment in Combination with the Herbal Medicine Black Cohosh

**DOI:** 10.3390/ijms242417506

**Published:** 2023-12-15

**Authors:** Agata Płoska, Marcin Wozniak, Jamila Hedhli, Christian J. Konopka, Antonios Skondras, Sarah Matatov, Andrew Stawarz, Sarah Schuh, Andrzej Czerwinski, Lawrence W. Dobrucki, Leszek Kalinowski, Iwona T. Dobrucki

**Affiliations:** 1 Department of Medical Laboratory Diagnostics-Fahrenheit Biobank BBMRI.Pl, Medical University of Gdansk, 80-211 Gdansk, Poland; agata.ploska@gumed.edu.pl (A.P.); marcin.wozniak@gumed.edu.pl (M.W.); dobrucki@illinois.edu (L.W.D.); 2 Beckman Institute for Advanced Science and Technology, Urbana, IL 61801, USA; hedhli2@illinois.edu (J.H.); ckonopka92@siumed.edu (C.J.K.);; 3 Carle-Illinois College of Medicine, University of Illinois at Urbana-Champaign, Urbana, IL 61801, USA; 4 Department of Bioengineering, University of Illinois at Urbana-Champaign, Urbana, IL 61801, USA; 5 Cancer Center at Illinois, University of Illinois at Urbana-Champaign, Urbana, IL 61801, USA; 6 BioTechMed Centre, Department of Mechanics of Materials and Structures, Gdansk University of Technology, 80-233 Gdansk, Poland; 7 Academy of Medical and Social Applied Sciences, 82-300 Elblag, Poland

**Keywords:** complementary and alternative medicine, black cohosh, doxorubicin, angiogenesis, α_v_β_3_ integrin, FDG, tumor metabolism

## Abstract

As a substitution for hormone replacement therapy, many breast cancer patients use black cohosh (BC) extracts in combination with doxorubicin (DOX)-based chemotherapy. In this study, we evaluated the viability and survival of BC- and DOX-treated MCF-7 cells. A preclinical model of MCF-7 xenografts was used to determine the influence of BC and DOX administration on tumor growth and metabolism. The number of apoptotic cells after incubation with both DOX and BC was significantly increased (~100%) compared to the control. Treatment with DOX altered the potential of MCF-7 cells to form colonies; however, coincubation with BC did not affect this process. In vivo, PET-CT imaging showed that combined treatment of DOX and BC induced a significant reduction in both metabolic activity (29%) and angiogenesis (32%). Both DOX and BC treatments inhibited tumor growth by 20% and 12%, respectively, and combined by 57%, vs. control. We successfully demonstrated that BC increases cytotoxic effects of DOX, resulting in a significant reduction in tumor size. Further studies regarding drug transport and tumor growth biomarkers are necessary to establish the underlying mechanism and potential clinical use of BC in breast cancer patients.

## 1. Introduction

Over the past few decades, complementary and alternative medicine (CAM) has visibly grown in popularity. In December 2008, the National Center for Complementary and Integrative Health (NCCIH) reported that approximately 38% of American adults and 12% of children used some type of CAM during the previous 12 months. To place these numbers into perspective, the NCCIH notes that in 2007 there were about 354 million more patient visits to alternative health care than to primary care providers [1]. Most of these users pay out-of-pocket, and the estimated total cost of herbal supplements increases yearly. In 2019 across all market channels, consumers spent approximately USD 9.602 billion on herbal supplements [2]. Importantly, CAM use among patients with cancer has long been higher than among the overall population, and a 2014 survey by Kang et al. reported that patients with breast cancer tend to employ CAM more often than patients with other types of tumors [3].

Herbal therapy was one of the CAM modalities that increased the most among patients after a breast cancer diagnosis. Black cohosh (BC, *Actaea racemosa*, formerly known as *Cimicifuga racemosa*), a plant native to North America, has been used for the last 60 years as a natural alternative to hormone replacement therapy and for other gynecological conditions. Women increasingly turn to BC as a “natural” alternative to estrogen replacement therapy. According to Gaffner, between 2012 and 2014, BC was one of the 10 top-selling herbs in the mainstream market and has ranked within the 30 top-selling herbs in the natural foods sector in the United States [4]. In the latest report from 2019, BC was in 15th place among the top-selling herbal supplements in U.S. Mainstream Multi-Outlet Channel [2]. The *Actaea* (or *Cimicifuga*) genus has a long history of use in traditional herbal medicine. *Cimicifuga dahurica*, *C. foetida*, and *C. heracleifolia* are three officially listed species in the Pharmacopoeia of the People’s Republic of China [5,6].

The traditional use of root and rhizome of BC in North America includes the management of ailments such as sore throats, colds, malaria, rheumatism, snakebite, complications associated with childbirth, and to stimulate menstruation [7,8]. BC was exported to Europe as early as the 18th century and used by Europeans to treat menopausal symptoms and other difficulties related to the female reproductive system [9,10]. Currently, BC is widely used to alleviate menopausal symptoms such as hot flashes, night sweats, sleep disturbances, vertigo, nervousness, mood swings, and vaginal dryness [10,11,12,13].

The first medicinal formulation of BC—an isopropanolic extract standardized to triterpene glycosides (Remifemin^®^)—has been available since 1956 [9]. Up to today, approximately 40 clinical trials, including different BC extracts and formulations, have been conducted [11,12]. Several trials showed no impact of BC on alleviating menopausal symptoms, but most of them revealed the potential of BC as the alternative for hormone replacement therapy in menopausal women with very few adverse side effects [9,11,12,14,15].

Currently, 131 bioactive species from BC rhizoma have been identified [11]. Among them are triterpene glycosides (e.g., 23-epi-26-deoxyactein—previously known as 27-deoxyactein, actein, cimiracemoside), phenolic compounds (e.g., caffeic acid, hydroxycinnamic acids, ferulic acid, isoferulic acid, cimicifugic acids), alkaloids (isoquinoline, indole, and guanidine-type), and related compounds that contain nitrogen (e.g., cimipronidine, dopargine, Nω-methylserotonin) [11,12,16].

The results of in vitro studies have demonstrated the anticancer effect of BC extract on various breast cancer cell lines. The mechanism responsible for the inhibitory effect of BC on MCF-7 and MDA-MB-231 breast cancer cells was either cycle arrest [17], activation of caspases and induction of apoptosis [18,19], or downregulation of estrogen receptor alpha expression in T-47D and MCF-7 hormone-dependent breast cancer cells [20]. Actein, an active substance isolated from BC extract, showed antimetastatic potential by reducing the proliferation, migration, and motility of MCF-7 and MDA-MB-231 cells [21] and decreasing tumor volume and metastasis in murine 4T1 breast cancer cells [22]. Other studies have demonstrated the sensitization effect of BC to doxorubicin and docetaxel treatment in murine EMT6 cells [23].

Nevertheless, little is still known about the in vivo metabolism of many of these compounds, and even less is known about their potential interaction with anticancer therapies.

Doxorubicin (DOX) is an anthracycline antibiotic that has been shown to promote tumor cell death primarily by the induction of apoptosis [24]. DOX was isolated from *Streptomyces peucetius* in the 1950s when it was discovered to inhibit tumor growth in mice. Liposomal DOX hydrochloride was one of the first liposome-encapsulated anticancer drugs to receive clinical approval for use against a few malignancies, including solid tumors, leukemia, and lymphomas [25]. The mechanism of action of DOX remains not fully understood, and there exist major concerns about its cardiotoxicity, most likely due to increased production of reactive oxygen species (ROS) [26,27].

This article investigates the effects of the simultaneous administration of commercially available BC extract and DOX in human breast cancer cells (MCF-7) and in the preclinical model of MCF-7 xenograft. To noninvasively quantify in vivo tumor metabolism and angiogenesis, we employed PET-CT imaging with ^18^F radiolabeled glucose analogue (fluorodeoxyglucose, FDG) and ^64^Cu-labeled dimeric cRGD peptide (^64^Cu-NOTA-PEG_4_-cRGD_2_) developed by our group, respectively.

## 2. Results

In the present study, we evaluated the effects of combined BC and DOX therapy on the MCF-7 cell line and employed radiolabeled tracers to noninvasively image the tumor microenvironment (both angiogenesis and metabolism) in a murine model of MCF-7 xenografts.

### 2.1. Results of In Vitro Studies

**Figure 1:**
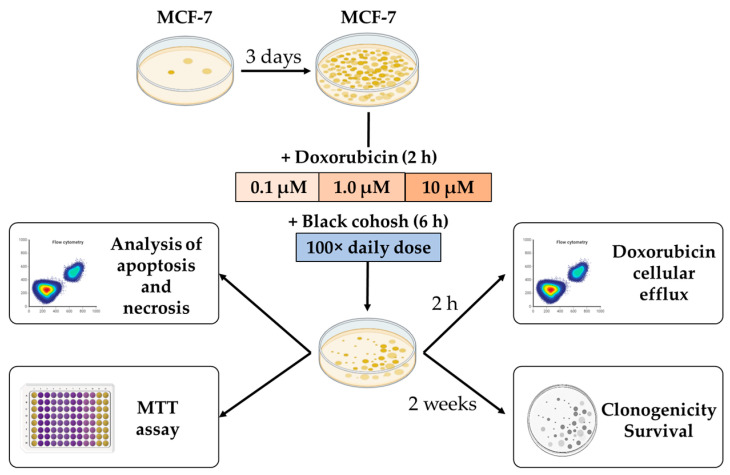
Schematic diagram of in vitro studies in MCF-7 adenocarcinoma cells. Cancer cells were cultured for 3 days to reach confluency and then incubated with various concentrations of DOX with or without the presence of BC extract. Following incubation, the amount of alive, apoptotic, and dead cells and cellular doxorubicin efflux were analyzed with flow cytometry. The viability of cells was measured with MTT assay, and the potential of MCF-7 cells to form colonies was studied with a clonogenic assay. Each result was obtained in triplicate.

MCF-7 cells were cultured and analyzed to determine the effect of DOX and BC treatment on survival, proliferation, the potential of MCF-7 to form colonies, and to assess BC’s influence on DOX efflux. The in vitro studies were performed according to the research plan outlined in Figure 1.

**Figure 2:**
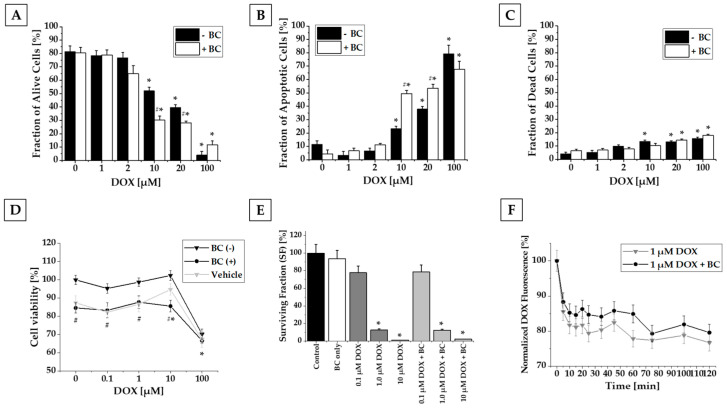
Results from in vitro studies. FC analysis of cell viability (**A**–**C**), MTT assay (**D**), clonogenic assay (**E**), and doxorubicin efflux study (**F**). Flow cytometry analysis of MCF-7 cells incubated with CaM and PI was performed to determine the amount of live (**A**), apoptotic (**B**), and dead (**C**) cells. Treatment with DOX at concentrations of 10 µM and higher resulted in significantly decreased number of alive cells and increased number of apoptotic and dead cells. Treatment with the combination of DOX with BC caused a similar effect. Additionally, for the combination of 10 and 20 µM DOX with BC, statistically significant differences were found compared to treatment with DOX alone. In MTT assay, DOX alone or in combination both with BC extract and vehicle significantly reduced a number of alive cells only at the highest concentration. BC and vehicle significantly decreased MCF-7 viability compared to treatment with DOX alone (**D**). The surviving fraction of MCF-7 cells in the clonogenic assay decreased after incubation with DOX alone or combined with BC, but there was no significant difference after addition of BC (**E**). FC analysis of DOX efflux after 2 h incubation with 1 µM DOX revealed that BC decreased the efflux of doxorubicin, but there were no significant differences (**F**). ((*) *p* < 0.05 compared to the control, (#*) *p* < 0.05 compared to the results for treatment with DOX alone, (#) *p* < 0.05 for both BC and vehicle compared to the results for treatment with DOX alone.

Flow cytometry analysis of alive, apoptotic, and dead cells revealed that 2 h incubation with DOX in concentrations higher than 10 µM reduced the number of alive and increased the number of apoptotic and dead MCF-7 cells in a concentration-dependent manner. A similar effect was observed after incubation of MCF-7 cells with a combination of DOX and BC. Interestingly, coincubation with BC resulted in a shift towards apoptosis. The combination of DOX (10 and 20 µM) and BC caused a significant decrease in alive (30% and 28% vs. DOX alone) and an increase in apoptotic (49% and 54% vs. DOX alone) cell fractions when compared to treatment with DOX only (Figure 2A–C).

MTT assay revealed that DOX either alone or in combination with BC or vehicle significantly reduced the number of alive MCF-7 cells only at the highest used concentration—100 µM (33% for combination of DOX with both BC and vehicle vs. 30% for DOX alone). Doxorubicin in lower concentrations had no impact on the number of MCF-7 alive cells. Interestingly, coadministration of BC or vehicle decreased the number of alive MCF-7 cells independently from DOX presence by about 15 and 12%, respectively (Figure 2D). No significant changes were observed for the lower doses (1× and 10× daily use) of BC extract.

Based on performed clonogenic assay, we demonstrated that treatment with DOX altered the MCF-7 cells’ survival and the potential to form colonies; nevertheless, there was no statistically significant difference between the cells treated with DOX only or with DOX and BC combined (Figure 2E).

Conducted flow cytometry analysis revealed that DOX efflux in MCF-7 cells incubated with both DOX and BC compared to DOX only slowed down, but there was no significant difference (Figure 2F).

### 2.2. Results of In Vivo Studies

**Figure 3:**
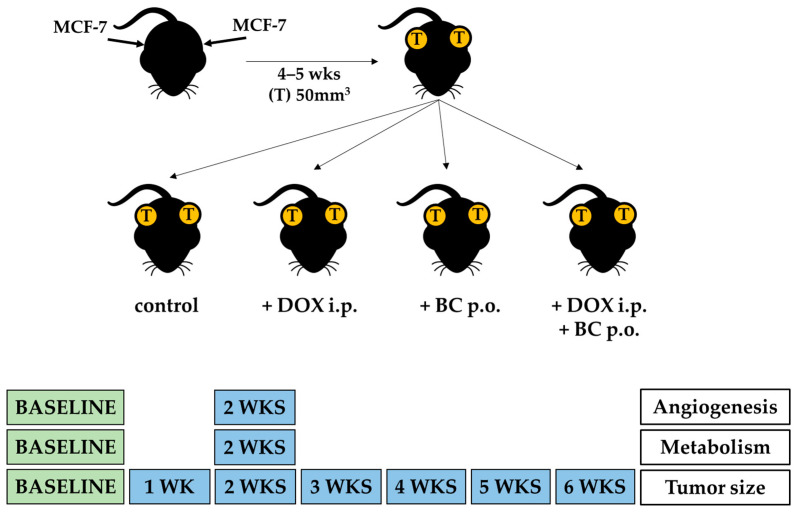
Schematic diagram of in vivo studies in a murine model of MCF-7 xenografts. Mice were injected with MCF-7 cells in both flanks and implanted with estradiol pellets to allow for tumor growth. After 4–5 weeks of tumor growth, animals were divided into four groups: control (no treatment) and those receiving DOX (4 mg/kg/wk, i.p.), BC (20 mg/kg/d, p.o.), or both DOX and BC combined. All mice were monitored daily, and tumor volume measurements were taken twice a week using digital calipers. To evaluate tumor metabolism and angiogenesis at baseline and 2 weeks after starting the treatment, microPET-CT imaging was performed using ^18^F-FDG and ^64^Cu-NOTA-PEG_4_-cRGD_2_, respectively.

The schematic diagram of in vivo experiments is presented in Figure 3. For in vivo studies, 21-week-old ovariectomized athymic nude mice were used. Briefly, all animals were injected with MCF-7 and implanted with estradiol pellets. After tumor growth exceeded 50 mm^3^ volume, mice were divided into four groups: control animals and those receiving DOX, BC, or both DOX and BC.

**Figure 4:**
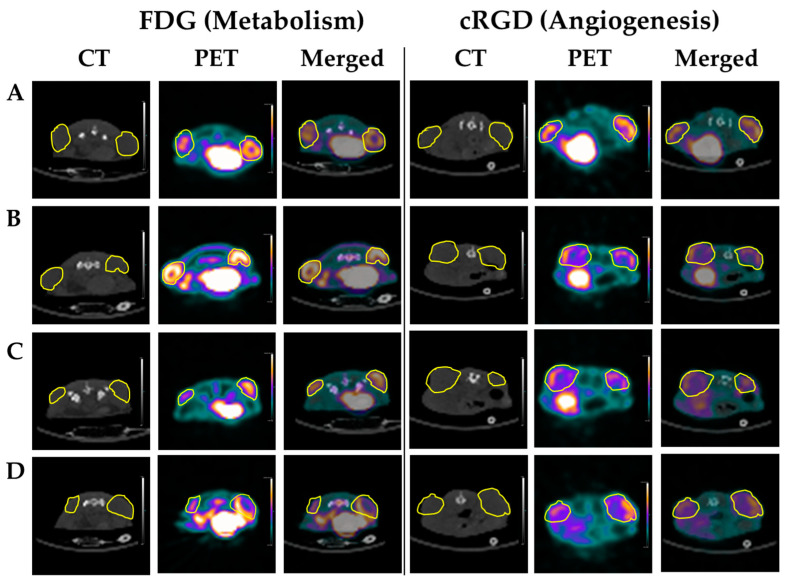
Representative PET-CT axial slices of FDG (**left**) and cRGD (**right**) imaging studies at 2 weeks for control (**A**), BC (**B**), DOX (**C**), and DOX + BC (**D**) treatment groups, with tumors circled in yellow.

Tumor-bearing animals from each treatment group were subjected to a baseline and a 2-week PET-CT imaging using either FDG (for metabolism) or cRGD (for angiogenesis) tracers. Representative PET-CT images of FDG and cRGD imaging studies are shown in Figure 4. Following the imaging and after tumor volumes-of-interest (VOIs) were defined and standardized uptake values (SUV) calculated, the differences between baseline and 2-week maximal SUV were examined.

**Figure 5:**
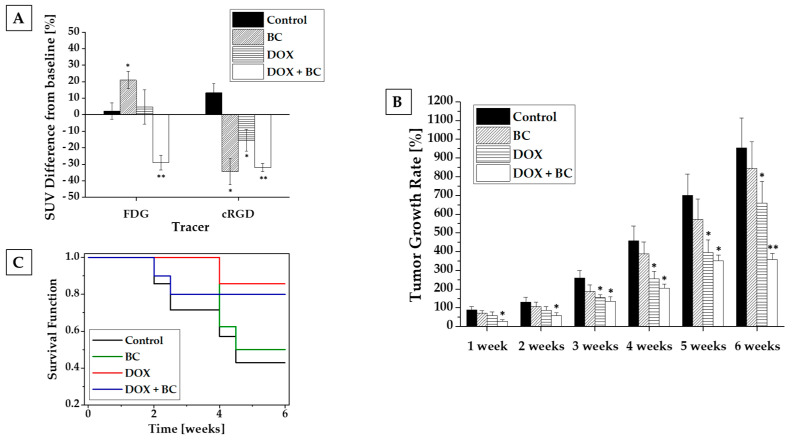
Results from in vivo studies. PET-CT imaging with FDG and cRGD (**A**), tumor growth rate (**B**), and Kaplan–Meier survival curves (**C**). Before and 2 weeks after implementation of the treatment, tumor-bearing animals from each treatment group were subjected to PET-CT imaging using either FDG (metabolism) or cRGD (angiogenesis) tracers. In imaging with FDG, BC caused a statistically significant increase in metabolic activity compared to the control group. Treatment with DOX did not change the tumor metabolism, but the combination of DOX and BC showed the greatest statistically significant reduction in metabolic activity. Imaging with cRGD showed a statistically significant reduction of angiogenesis in all treated groups compared to the control group (**A**). A combination of BC and DOX resulted in a significant reduction in tumor size observed since the first week of treatment compared to the control group. During the last measurements, at 6 weeks of treatment, a statistically significant difference in tumor growth rate was observed between the group treated with combination of DOX and BC compared to the DOX-only-treated group (**B**). The analysis of survival function demonstrated no significant differences between experimental groups (**C**). (*) *p* < 0.05 compared to the control group. (**) *p* < 0.05 compared both to the control group and group treated with DOX alone.

The BC treatment group showed a 20% increase in metabolic activity (FDG imaging) compared to the control group. However, a significant 33% decrease in integrin expression suggests decreased angiogenesis (cRGD imaging). Doxorubicin-treated animals demonstrated no change in metabolic activity from baseline imaging but a significant decrease of 16% in angiogenesis. The combined treatment of DOX and BC showed the greatest statistically significant reduction in both metabolic activity (29%) and angiogenesis (32%), which strongly supports the hypothesis that BC potentiates the therapeutic effect of DOX on MCF-7 xenografts (Figure 5A). Furthermore, BC enhances the cytotoxic effects of DOX, resulting in a significant reduction in tumor size observed as early as the second week of treatment. Tumor volume measurements during the sixth week of therapy demonstrated that combined DOX and BC treatment resulted in a 57% reduction in tumor size compared to DOX-only treated animals (Figure 5B). Analysis of survival during the treatment revealed no statistically significant differences between experimental groups. However, the higher survival rate was observed for animals receiving treatment with DOX (Figure 5C).

### 2.3. Results of Ex Vivo Studies

**Figure 6:**
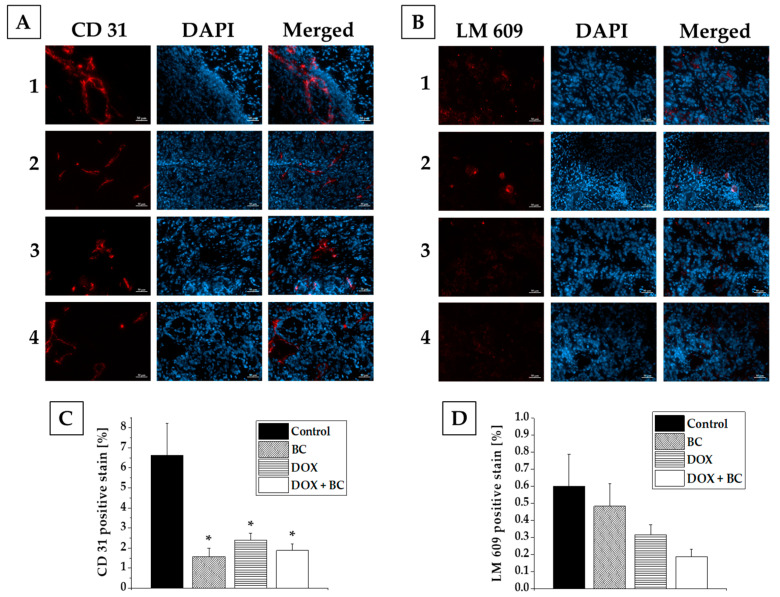
Representative immunofluorescence images obtained from MCF-7 xenografts isolated from mice. After 6 weeks of the treatment with BC, DOX, or a combination of DOX and BC, animals were sacrificed, tumors excised, sectioned, and stained for CD31 (**A**) and LM609 (**C**). The expression of CD31 (endothelial marker) was significantly lower in all treated groups of animals (BC, DOX, DOX + BC) compared to the control group (**B**). The expression of LM609 (α_v_β_3_ integrins) was decreased both in DOX only and DOX with BC treated group compared to the control group, but with no statistical relevance (**D**). The immunostaining showed no statically significant differences between the groups treated with DOX only and the combination of DOX with BC. Scale bar: 50 µm, (*) *p* < 0.05 compared to the control group.

To investigate the impact of DOX and BC treatment at the cellular level, 6 weeks after the beginning of the treatment, animals were euthanized, and tumors were excised and stained for CD31 (endothelial cell marker) and LM609 (α_v_β_3_ integrins, biomarkers for angiogenesis). Subsequent fluorescence microscopic imaging showed a statistically significant decrease in the CD31 expression (4.2, 2.8, and 3.5-fold for BC, DOX, and DOX + BC, respectively, in comparison to the nontreated control group) for all treated groups (Figure 6A,C). Also, tumor angiogenic activity decreased after treatment (1.2, 1.9, and 3.2-fold for BC, DOX, and DOX + BC, respectively, in comparison to the nontreated control group), but there was no statistical relevance (Figure 6B,D). For both types of staining, the additional effect of BC on DOX therapy was observed, especially in angiogenesis, but there was no significant difference between the DOX and DOX + BC treated groups. Data obtained from fluorescence microscopic imaging confirmed the results from microPET-CT imaging of angiogenesis.

## 3. Discussion

The use of complementary and alternative medicine (CAM) has seen a significant increase in popularity in recent years, with a quarter of adults in the Western world using some form of herbal medicine as a supplement to their traditional treatments [28]. This trend has placed a significant burden on the healthcare system; conversely, there is currently a limited understanding of the interaction between CAM and conventional medical treatments.

Patients, especially those with cancer, often choose to supplement their prescribed therapies with natural medicines. This choice is often made in an effort to alleviate the stress and anxiety associated with a cancer diagnosis or to explore all possible treatment options [29]. Studies have shown that a majority of cancer patients, particularly those with breast, prostate, and skin cancer, use plant-derived drugs alongside their chemotherapy drugs during the early stages of pharmacotherapy [30,31]. Therefore, there is an unmet need to study the effectiveness and safety of all therapies (both CAMs and non-CAMs).

BC is a well-known herbal remedy that has been shown to be effective in a number of clinical trials. It has been shown to be helpful in reducing menopausal symptoms, decreasing anxiety, and improving mood symptoms, quality of sleep, bone density, and metabolism [9,11,12,32]. Additionally, it has been reported to have a positive impact on increasing the pregnancy rate in women with polycystic ovary syndrome (PCOS) [33]. These studies support the use of BC as a valuable treatment option for a variety of health issues.

Einbond et al. reported that BC extract inhibited the growth of MCF-7 human breast cancer cells due to cycle arrest, and this inhibition was increased by following transfection of the cells with Her2 [17]. Rice et al. and Hostanska al. showed that BC significantly inhibited the growth of both MCF-7 and MDA-MB-123 cells due to the activation of caspases and induction of apoptosis [18,19]. Also, actein isolated from BC proved to be a potential antimetastatic candidate for treating advanced human breast cancer [21]. Antiproliferative and proapoptotic gene expression induced in MCF-7 cells after incubation with BC was shown at the transcriptional level [34]. The cytotoxic effect of BC extracts was also confirmed in other cancer cells. Hostanska et al. demonstrated BC growth-inhibitory and apoptotic potential on various prostate carcinoma cells representing different developmental stages and androgen responsiveness (LNCaP, DU145, and PC-3) [35]. Actein isolated from BC significantly inhibited the colony formation ability of 143B and U2OS osteosarcoma cells. Additionally, the viability of 143B osteosarcoma cells was inhibited after actein treatment in both a time- and dose-dependent manner [36]. Jöhrer et al. revealed the cytotoxic effect of several cimigenol-type triterpenoids isolated from BC on multiple myeloma cell lines (NCI-H929, OPM-2, and U266) [37]. Actein also suppressed cell proliferation and induced autophagy and apoptosis in human bladder cancer [38].

Although some studies in mouse mammary cell lines suggest that BC may sensitize cells and enhance the actions of certain chemotherapeutic drugs, the interaction of BC with doxorubicin (DOX, the most widely prescribed anticancer drug) remains unclear. Several studies focus on the influence of extracts from *Cimicifuga* sp. on drug efflux and transporting proteins. Rockwell et al. focused on the influence of addition of BC extracts to cancer therapy and showed that BC increases the cytotoxicity of doxorubicin in murine EMT6 cells due to changes in drug efflux [23]. Acerinol isolated from *C. acerina* can potentially reverse the ABCB1-mediated multidrug resistance (MDR) in cancer cells. The responsible mechanism involves inhibiting the efflux function of ABCB1 and increasing intracellular retention of anticancer drugs [39]. Sinreih et al. demonstrated that very high levels of BC affected the expression of genes encoding estrogen E1-S transporters and estrogen-related enzymes [40].

In the present study, we investigated both in vitro and in vivo synergistic effects of herbal medicine, black cohosh (BC), and conventional anticancer therapeutics, doxorubicin (DOX), on the tumor microenvironment of MCF-7 cells.

We began our investigations by studying the effect of DOX and BC on survival and proliferation rate. In the MTT assay, DOX decreased the number of alive MCF-7 cells only at the highest concentration (100 µM). The cytotoxic effect of BC extract seemed to be caused by vehicle (ethanol), not BC alone. Treatment with DOX altered the MCF-7 cells’ survival, proliferation, and the potential for MCF-7 to form colonies, but there was no significant difference between cells treated with DOX only or DOX and BC combined in the colony-forming assay (Figure 2E). The flow cytometry experiments demonstrated that DOX concentrations higher than 10 µM alone or in combination with BC decreased the number of alive cells and increased the number of apoptotic and dead cells. Worthy of mention is the fact that the addition of BC to incubation with DOX in 10 and 20 µM concentrations resulted in increased apoptosis, enhancing the cytotoxic effect of DOX (Figure 2A–C).

Based on flow cytometry analysis, we observed decreased efflux in DOX efflux from MCF-7 cells incubated with both DOX and BC compared to DOX alone, but with no significant difference (Figure 2F).

Our study findings appear to slightly contradict those previously reported in the literature. For example, Rockwell et al. demonstrated the effect of BC extract, doxorubicin, cisplatin, docetaxel, and cyclophosphamide on murine breast cancer cell colonies [23]. They observed increased cellular cytotoxicity of doxorubicin and docetaxel in the presence of BC extract, as well as reduced efficacy of cisplatin in the presence of BC. Moreover, they also found that the treatment with both doxorubicin and BC resulted in a 40-fold lower survival rate of cancer cells compared to cells incubated with doxorubicin alone. However, it is important to note that the discrepancy between our findings and Rockwell’s study may be due to the use of different cancer cell lines (murine vs. human) and distinct BC extracts utilized by our groups. The BC extracts used by Rockwell were standardized and contained a specific amount of 180 active ingredients (the first 3% triterpene glycosides, the second 2.5%, and the third 2 mg terpene glycodeoxyactein and 1 mg isoflavones) [23]. Our studies employed the BC extract, obtained from commercially available supplement, with the final concentration of triterpene glycosides equal to 2.5 mg per 1 mL of solution.

One potential explanation for the differences in results presented in our study compared to other reports is that the composition of the BC extract used in our studies is not precisely defined. This means that, although the BC extract was standardized to triterpene glycosides, the exact characteristic and proportion of compounds present in the extract that we used may differ from other studies. However, this decision was made intentionally considering the high availability of plant-based medicines and the general perception that they are safe. It is worth noting that the composition of an extract is a crucial aspect that can greatly influence the observed effect. Different solvents used for extraction result in different compositions and concentrations of the active ingredient in available BC supplements. Additionally, a great number of BC formulations commercially available in the U.S. are adulterated [15,41,42,43,44].

The other possible explanation for this discrepancy could be the relatively short incubation time. In our study, MCF-7 cells were incubated with DOX for only 2 h, proceeded by 6 h of incubation with BC extract. All studies included a longer period (12–96 h) for incubation, both with DOX and BC [17,18,19,20,21,23,34,35,36,37,38,40]. Additionally, the concentration of BC extract used in our studies, equal to 100x the recommended daily dose, was still much lower (10.4 µg/mL) then the toxic concentration for MCF-7 cells reported by Hostanska et al. (CC50 = 80.6 µg/mL for ethanolic BC extract) [19].

Although in our in vitro studies we were not able to prove the synergism of BC with DOX on MCF-7 cells, our in vivo research did show significant advantages when BC was administered together with DOX. The PET-CT imaging revealed that the addition of BC to DOX treatment resulted in decreased angiogenesis. The antiangiogenic potential of actein isolated from BC was also confirmed previously by Yue et al. [22]. The specific mechanism involves the suppression of the VEGFR1, pERK, pINK, and JNK/ERK pathways. Also, downregulation of the expression of VEGFR1 and CXCR4 was shown in breast cancer [22].

In our studies, the combination of DOX and BC was able to increase metabolism and decrease angiogenesis, as confirmed by immunohistological staining, which in turn leads to a significant reduction in tumor growth in a murine xenograft model. The benefits of BC addition to DOX treatment resulting in decreased tumor growth were clearly visible, especially after long-term therapy.

The results of our study showed the potential of BC extract for use in combined anticancer therapy with DOX. To fully understand the mechanism by which BC extract restricts the growth of breast tumors and to allow the future use of BC in anticancer treatment, further research is needed, including experiments on noncancer cells and animals, both on toxicology and time- and dose-dependent effect of BC extract on various breast cancer cell lines. Future experiments, including studies using standardized BC extracts with the exact composition of the BC extract used, are required. However, it has been determined that BC treatment is relatively safe. As a result, BC extract can be considered an adjuvant therapy, potentially contributing to the inhibition of tumor growth in anticancer treatments.

## 4. Materials and Methods

### 4.1. In Vitro Studies

The research plan for in vitro studies is outlined in Figure 1. Briefly, MCF-7 cells were cultured for 3 days and then incubated with various concentrations of DOX with or without the presence of BC extract. BC extract was obtained from a commercially available product: “Black Cohosh Single Herb Extract” capsules (Gaia Herbs Farm, Brevard, NC, USA). Each capsule contained 400 mg of BC root extract standardized to triterpene glycosides (2 mg per capsule). The content of 25 capsules was dissolved in 10 mL of 70% aqueous ethanol solution and ultracentrifuged to remove the remains of the capsule shell. The resulting solution was transferred to a volumetric flask and diluted with 70% aqueous ethanol to 20 mL. The prepared BC extract with a concentration of 500 mg/mL was filtered through a syringe filter (0.22 µm pore size) and stored at room temperature in a tightly closed dark bottle. The amount of BC extract for experiments was calculated based on recommended consumption for human use (30 to 40 drops; Gaia Herbs Farm, Brevard, NC, USA), standard human weight (70 kg) and average mouse parameters (25 g of body weight and 2 mL of blood volume).

#### 4.1.1. Cell Culture

The estrogen-sensitive human breast cancer cell line (Michigan Cancer Foundation-7, MCF-7) was obtained from American Type Culture Collection (ATCC, Gaithersburg, MD, USA) and grown to confluence in low-glucose Dulbecco’s minimum essential medium (DMEM) supplemented with 10% fetal bovine serum (GIBCO, Grand Island, NY, USA), 1% L-glutamine, 100 U/mL penicillin, 100 mg/mL streptomycin (GIBCO, Grand Island, NY, USA), 2.5 mg/mL amphotericin (Sigma-Aldrich, St. Louis, MO, USA), and 100 mg/mL gentamicin (GIBCO, Grand Island, NY, USA). The cells were maintained in a humidified incubator at 37 °C with 95% air and 5% CO_2_.

#### 4.1.2. Cell Viability Studies

To study the effect of BC and DOX on cell viability, an MTT assay was performed. Briefly, MCF-7 cells were seeded onto a 96-well plate at a density of 10^5^ cells per well and allowed to grow until confluency under standard culture conditions. The cells were then incubated with different concentrations of DOX (0.1, 1, 10, and 100 µM for 2 h) alone or combined with BC (20.8, 2.1, and 0.2 µL of BC extract per each mL of medium, which equals 100×, 10×, and 1× of the daily dose, for 6 h). The vehicle group was treated with medium containing 70% ethanol solution in the amount equal to added volume of BC extract (20.8, 2.1, and 0.2 µL/mL of medium). The control cells were treated with the medium alone (100% cell viability). After treatment, the cell culture medium was replaced with a fresh medium containing MTT (3-[4,5-dimethylthiazol-2-yl]-2,5-diphenyltetrazolium bromide, M2128, Sigma-Aldrich, St. Louis, MO, USA) at the final concentration of 0.5 mg/mL. After 4 h incubation at 37 °C, the medium with MTT was gently removed, and anhydrous DMSO (100 μL/well) was added to dissolve the formazan crystals. Absorbance was read using a BioTek Take3 microplate reader (BioTek Instruments, Inc., Winooski, VT, USA) at 540 nm. The data are presented as a percentage of the control.

#### 4.1.3. Flow Cytometric Analysis of Apoptosis and Necrosis

To study the effect of BC and DOX on cell viability, MCF-7 cells were plated onto 25 cm^2^ flasks (Corning Incorporated, Tewksbury, MA, USA) at a density of 10^6^ cells per flask and allowed to grow until confluency in standard culture conditions. The cells were then incubated with different concentrations of DOX (1, 2, 10, 20, and 100 µM for 2 h) alone or combined with BC (100× the daily dose for 6 h). Following the treatment phase, cells were lifted using accutase (Sigma-Aldrich, St. Louis, MO, USA), washed twice with PBS, resuspended in FACS buffer (5% FBS, 0.1% sodium azide in PBS), and incubated at 37 °C for 15 min with calcein-AM (CaM; 1 µM) and propidium iodide (PI; 2.5 µM) to distinguish live, apoptotic, and dead cells, respectively. Cells were then washed twice with PBS, resuspended in FACS buffer, and run on an LSR Fortessa (BD Biosciences, Franklin Lakes, NJ, USA) to perform flow cytometry analysis.

#### 4.1.4. Cell Survival Studies

MCF-7 cells were plated onto 25 cm^2^ flasks (Corning Incorporated, Tewksbury, MA, USA) at a density of 10^6^ cells per flask and allowed to grow in standard culture conditions until confluency. The cells were then incubated with different concentrations of DOX (0.1, 1, and 10 µM for 2 h) alone or combined with BC (100× the daily dose for 6 h). Following the treatment phase, cells were detached using accutase, counted on a Multisizer Coulter Counter (Beckman Coulter, Indianapolis, IN, USA), and plated onto 60 mm Petri dishes containing 5 mL of medium at a density of 200 and 1000 cells per dish. The cells were cultured for 2 weeks to allow the formation of large colonies. After 2 weeks, the MCF-7 cells were fixed and stained with 0.5% crystal violet, and the resulting colonies were scanned with a Leica digital stereoscope and counted using ImageJ software version 1.54f.

#### 4.1.5. Doxorubicin Efflux Studies

To study the effect of BC on DOX efflux, MCF-7 cells were detached using accutase, washed twice with PBS, resuspended in FACS buffer (5% FBS, 0.1% sodium azide in PBS), and incubated with 1 µM DOX for 2 h and BC (100× the daily dose for 6 h) alone or combined. Additionally, cells were stained with PI (2.5 µM, 37 °C for 15 min) to distinguish live and dead cells, respectively. Cells were then washed twice with PBS and resuspended in FACS buffer to perform flow cytometry analysis. Cells were then run on an LSR Fortessa (BD Biosciences, Franklin Lakes, NJ, USA) to evaluate DOX efflux.

### 4.2. In Vivo Studies

All in vivo experiments were performed with the approval of the Institutional Animal Care and Use Committee (IACUC) of the University of Illinois at Urbana-Champaign, following the principles outlined by the American Physiological Society on research animal use. All animal work was performed in accordance with the guidelines and best practices outlined by the IACUC and ARRIVE.

The 21-week-old ovariectomized athymic nude mice were injected with MCF-7 cells in both flanks and implanted with estradiol pellets to allow for tumor growth. All mice were monitored daily for the presence of tumors. When the tumors became palpable, volume measurements were taken twice a week using digital calipers (Absolute Solar Digimatic, Mitutoyo America Corporation, Aurora, IL, USA). The volume was calculated using a simplified approximation formula:

$$Volume=\frac{{length\times width}^{2}}{2}$$

After tumor growth exceeded 50 mm^3^ volume (4 to 5 weeks), animals were divided into four groups: a control (no treatment, n = 7) and those receiving DOX (4 mg/kg/week, n = 7), BC (20 mg/kg/day, n = 8), or both DOX and BC (n = 10). The BC dose was equal to recommended daily dose for human use. After 2 weeks of treatment, all animals underwent PET-CT imaging for tumor metabolism and angiogenesis. After 6 weeks of treatment, animals were sacrificed, and tissues harvested. The schematic diagram of in vivo experiments is presented on Figure 3.

#### 4.2.1. In Vivo Imaging of MCF-7 Xenografts

To evaluate tumor metabolism and angiogenesis microPET-CT imaging was performed using ^18^F-FDG (FDG) and ^64^Cu-NOTA-PEG_4_-cRGD_2_ (cRGD) developed by our group [45,46], respectively, at baseline, as well as 2 weeks after starting the treatment (Figure 3). All animals were anesthetized with 1–3% isoflurane, the neck area was shaved, and the left jugular vein was isolated to facilitate the injection of the radiotracers. The animals, after overnight fasting, were injected with approximately 29.6 MBq of ^18^F-FDG or ^64^Cu labeled cRGD on alternative days. Imaging was performed using a hybrid small animal microPET-CT scanner (Inveon, Siemens Healthcare, Malvern, PA, USA). Following the administration of radiotracers, mice were placed on an animal bed, and at 30 min (FDG) or 60 min (cRGD) after radiotracer injection, a 15 min microPET imaging was performed. This was followed by high-resolution anatomical micro CT imaging (360 projections, 80 keV, and 500 mA energy).

#### 4.2.2. Image Analysis

PET-CT images of tumor-bearing animals were analyzed using Inveon Research workplace. Volumes-of-interest (VOIs) were created by manually tracing tumors on CT datasets, which were clearly visible, with numerous 2-D axial ROIs, followed by interpolation of those 2-D regions to yield the tumor VOIs. No partial volume correction was applied as tumors were a volume significantly larger than the system’s spatial resolution. Volume and mean percent injected dose per gram tissue (% ID/g) were then calculated. Standard uptake values (SUVs) were then calculated for each tumor.

### 4.3. Ex Vivo Studies

After 6 weeks of treatment, mice were sacrificed and tissues were harvested. Tumor samples were embedded in the Optimal Cutting Temperature (OCT) compound (TissueTec, Sakura, Torrance, CA, USA) and snap-frozen in liquid nitrogen. Tissues were cut into 5 µm sections using a cryotome. The sections were fixed in ice-cold acetone and stained with either an endothelial cell-specific marker: PE-labeled anti-mouse CD31 Antibody (Abcam, Waltham, MA, USA) or a commercially available α_V_β_3_ marker: PE-LM609 (R&D Systems, Minneapolis, MN, USA). The sections were incubated with antibodies (1:50 dilution in PBS) for 1 h in a wet chamber at room temperature. After incubation with the antibodies, the stained sections were mounted with Fluoro-Gel II Mounting Medium containing DAPI (Electron Microscopy Sciences, Hatfield, PA, USA). Fluorescent images were acquired using a Zeiss Axiovert 200M microscope (Carl Zeiss, Oberkochen, Germany) equipped with 10× and 20× objectives. Several randomly chosen fields (200×) from each acquired image were processed with Zeiss Zen Blue software version 3.7 to calculate the total positively stained area.

### 4.4. Statistical Analysis

The Student’s *t*-test was used to compare the results between the two groups. One-way ANOVA was used to compare multiple parameters. Survival up to 6 weeks of treatment was shown as a Kaplan–Meier survival curve. A value of *p* < 0.05 was considered significant.

## Data Availability

Raw data were generated at the Molecular Imaging Laboratory, Biomedical Imaging Center at Beckman Institute for Advanced Science and Technology on the University of Illinois at the Urbana-Champaign campus. Derived data supporting the findings of this study are available from the corresponding author (I.T.D.) on request. The authors confirm that the data supporting the findings of this study are available within the article.

## References

[1] Barnes P.M., Bloom B., Nahin R.L. (2008). Complementary and alternative medicine use among adults and children: United States, 2007. Natl. Health. Stat. Report.

[2] Smith T., May G., Eckl V., Reynolds C.M. (2020). US sales of herbal supplements increase by 8.6% in 2019. Herb. Gram..

[3] Kang D.H., McArdle T., Suh Y. (2014). Changes in complementary and alternative medicine use across cancer treatment and relationship to stress, mood, and quality of life. J. Altern. Complement. Med..

[4] Gafner S. (2016). Botanical Adulterants Bulletin on Adulteration of *Actaea racemosa*. Botanical Adulterants Bulletin. United States. http://cms.herbalgram.org/BAP/pdf/BAP-BABs-BlackCohosh-FINAL.pdf.

[5] Guo Y., Yin T., Wang X., Zhang F., Pan G., Lv H., Wang X., Owoicho Orgah J., Zhu Y., Wu H. (2017). Traditional uses, phytochemistry, pharmacology and toxicology of the genus Cimicifuga: A review. J. Ethnopharmacol..

[6] Li J.X., Yu Z.Y. (2006). Cimicifugae rhizoma: From origins, bioactive constituents to clinical outcomes. Curr. Med. Chem..

[7] Setzer W.N. (2018). The Phytochemistry of Cherokee Aromatic Medicinal Plants. Medicines.

[8] Kenda M., Glavač N.K., Nagy M., Sollner Dolenc M., On Behalf of The Oemonom (2021). Herbal Products Used in Menopause and for Gynecological Disorders. Molecules.

[9] Henneicke-von Zepelin H.H. (2017). 60 years of Cimicifuga racemosa medicinal products: Clinical research milestones, current study findings and current development. Wien. Med. Wochenschr..

[10] Predny M.L., De Angelis P., Chamberlain J.L. (2006). Black Cohosh (*Actaea racemosa*): An Annotated Bibliography. U.S. Department of Agriculture Forest Service, Southern Research Station, General Technical Report SRS 97. United States. https://www.srs.fs.usda.gov/pubs/gtr/gtr_srs097.pdf.

[11] Salari S., Amiri M.S., Ramezani M., Moghadam A.T., Elyasi S., Sahebkar A., Emami S.A. (2021). Ethnobotany, Phytochemistry, Traditional and Modern Uses of *Actaea racemosa* L. (Black cohosh): A Review. Adv. Exp. Med. Biol..

[12] Mohapatra S., Iqubal A., Ansari M.J., Jan B., Zahiruddin S., Mirza M.A., Ahmad S., Iqbal Z. (2022). Benefits of Black Cohosh (*Cimicifuga racemosa*) for Women Health: An Up-Close and In-Depth Review. Pharmaceuticals.

[13] (2018). Black Cohosh Herbal Summary. European Medicines Agency. https://www.ema.europa.eu/en/documents/herbal-summary/black-cohosh-summary-public_en.pdf.

[14] Naser B., Schnitker J., Minkin M.J., de Arriba S.G., Nolte K.U., Osmers R. (2011). Suspected black cohosh hepatotoxicity: No evidence by meta-analysis of randomized controlled clinical trials for isopropanolic black cohosh extract. Menopause.

[15] Foster S. (2013). Exploring the Peripatetic Maze of Black Cohosh Adulteration: A Review of the Nomenclature, Distribution, Chemistry, Market Status, Analytical Methods and Safety. Herbal. Gram.

[16] Nikolić D., Lankin D.C., Cisowska T., Chen S.N., Pauli G.F., van Breemen R.B. (2015). Nitrogen-Containing Constituents of Black Cohosh: Chemistry, Structure Elucidation, and Biological Activities. Recent. Adv. Phytochem..

[17] Einbond L.S., Shimizu M., Xiao D., Nuntanakorn P., Lim J.T., Suzui M., Seter C., Pertel T., Kennelly E.J., Kronenberg F. (2004). Growth inhibitory activity of extracts and purified components of black cohosh on human breast cancer cells. Breast Cancer Res. Treat..

[18] Rice S., Amon A., Whitehead S.A. (2007). Ethanolic extracts of black cohosh (*Actaea racemosa*) inhibit growth and oestradiol synthesis from oestrone sulphate in breast cancer cells. Maturitas.

[19] Hostanska K., Nisslein T., Freudenstein J., Reichling J., Saller R. (2004). Evaluation of cell death caused by triterpene glycosides and phenolic substances from Cimicifuga racemosa extract in human MCF-7 breast cancer cells. Biol. Pharm. Bull..

[20] Crone M., Hallman K., Lloyd V., Szmyd M., Badamo B., Morse M., Dinda S. (2019). The antiestrogenic effects of black cohosh on BRCA1 and steroid receptors in breast cancer cells. Breast Cancer (Dove Med. Press).

[21] Wu X.X., Yue G.G., Dong J.R., Lam C.W., Wong C.K., Qiu M.H., Lau C.B. (2018). Actein Inhibits the Proliferation and Adhesion of Human Breast Cancer Cells and Suppresses Migration in vivo. Front. Pharmacol..

[22] Yue G.G., Xie S., Lee J.K., Kwok H.F., Gao S., Nian Y., Wu X.X., Wong C.K., Qiu M.H., Lau C.B. (2016). New potential beneficial effects of actein, a triterpene glycoside isolated from *Cimicifuga* species, in breast cancer treatment. Sci. Rep..

[23] Rockwell S., Liu Y., Higgins S.A. (2005). Alteration of the effects of cancer therapy agents on breast cancer cells by the herbal medicine black cohosh. Breast Cancer Res. Treat..

[24] Evan G.I., Vousden K.H. (2001). Proliferation, cell cycle and apoptosis in cancer. Nature.

[25] Slingerland M., Guchelaar H.J., Gelderblom H. (2012). Liposomal drug formulations in cancer therapy: 15 years along the road. Drug Discov. Today..

[26] Rivankar S. (2014). An overview of doxorubicin formulations in cancer therapy. J. Cancer. Res. Ther..

[27] Deng S., Kruger A., Kleschyov A.L., Kalinowski L., Daiber A., Wojnowski L. (2007). Gp91phox-containing NAD(P)H oxidase increases superoxide formation by doxorubicin and NADPH. Free Radic. Biol. Med..

[28] Cheng Y.Y., Hsieh C.H., Tsai T.H. (2018). Concurrent administration of anticancer chemotherapy drug and herbal medicine on the perspective of pharmacokinetics. J. Food Drug. Anal..

[29] Seely D., Oneschuk D. (2008). Interactions of natural health products with biomedical cancer treatments. Curr. Oncol..

[30] Pourroy B., Letellier C., Helvig A., Chanet B., De Crozals F., Alessandra C. (2017). Development of a rapid risk evaluation tool for herbs/drugs interactions in cancer patients: A multicentric experience in south of France. Eur. J. Cancer Care.

[31] Alsanad S.M., Howard R.L., Williamson E.M. (2016). An assessment of the impact of herb-drug combinations used by cancer patients. BMC Complement. Altern. Med..

[32] Wuttke W., Jarry H., Haunschild J., Stecher G., Schuh M., Seidlova-Wuttke D. (2014). The non-estrogenic alternative for the treatment of climacteric complaints: Black cohosh (*Cimicifuga* or *Actaea racemosa*). J. Steroid. Biochem. Mol. Biol..

[33] Arentz S., Abbott J.A., Smith C.A., Bensoussan A. (2014). Herbal medicine for the management of polycystic ovary syndrome (PCOS) and associated oligo/amenorrhoea and hyperandrogenism; a review of the laboratory evidence for effects with corroborative clinical findings. BMC Complement. Altern. Med..

[34] Gaube F., Wolfl S., Pusch L., Kroll T.C., Hamburger M. (2007). Gene expression profiling reveals effects of *Cimicifuga racemosa* (L.) NUTT. (black cohosh) on the estrogen receptor positive human breast cancer cell line MCF-7. BMC Pharmacol..

[35] Hostanska K., Nisslein T., Freudenstein J., Reichling J., Saller R. (2005). Apoptosis of human prostate androgen-dependent and -independent carcinoma cells induced by an isopropanolic extract of black cohosh involves degradation of cytokeratin (CK) 18. Anticancer. Res..

[36] Chen Z., Wu J., Guo Q. (2016). Actein Inhibits Cell Proliferation and Migration in Human Osteosarcoma. Med. Sci. Monit..

[37] Jöhrer K., Stuppner H., Greil R., Çiçek S.S. (2020). Structure-Guided Identification of Black Cohosh (*Actaea racemosa*) Triterpenoids with In Vitro Activity against Multiple Myeloma. Molecules.

[38] Ji L., Zhong B., Jiang X., Mao F., Liu G., Song B., Wang C.Y., Jiao Y., Wang J.P., Xu Z.B. (2017). Actein induces autophagy and apoptosis in human bladder cancer by potentiating ROS/JNK and inhibiting AKT pathways. Oncotarget.

[39] Liu D.L., Li Y.J., Yao N., Xu J., Chen Z.S., Yiu A., Zhang C.X., Ye W.C., Zhang D.M. (2014). Acerinol, a cyclolanstane triterpenoid from Cimicifuga acerina, reverses ABCB1-mediated multidrug resistance in HepG2/ADM and MCF-7/ADR cells. Eur. J. Pharmacol..

[40] Sinreih M., Gregorič K., Gajser K., Rižner T.L. (2022). Physiological Concentrations of Cimicifuga racemosa Extract Do Not Affect Expression of Genes Involved in Estrogen Biosynthesis and Action in Endometrial and Ovarian Cell Lines. Biomolecules.

[41] Pochet S., Lechon A.S., Lescrainier C., De Vriese C., Mathieu V., Hamdani J., Souard F. (2022). Herb-anticancer drug interactions in real life based on VigiBase, the WHO global database. Sci. Rep..

[42] Masada S. (2016). Authentication of the botanical origin of Western herbal products using Cimicifuga and Vitex products as examples. J. Nat. Med..

[43] Yu Y., Tan J., Nie J., Lv C., Lu J. (2022). Fibrous Roots of Cimicifuga Are at Risk of Hepatotoxicity. Molecules.

[44] Jiang B., Ma C., Motley T., Kronenberg F., Kennelly E.J. (2011). Phytochemical fingerprinting to thwart black cohosh adulteration: A 15 Actaea species analysis. Phytochem. Anal..

[45] Hedhli J., Czerwinski A., Schuelke M., Płoska A., Sowinski P., Hood L., Mamer S.B., Cole J.A., Czaplewska P., Banach M. (2017). Synthesis, Chemical Characterization and Multiscale Biological Evaluation of a Dimeric-cRGD Peptide for Targeted Imaging of α V β 3 Integrin Activity. Sci. Rep..

[46] Hedhli J., Slania S.L.L., Płoska A., Czerwinski A., Konopka C.J., Wozniak M., Banach M., Dobrucki I.T., Kalinowski L., Dobrucki L.W. (2018). Evaluation of a dimeric-cRGD peptide for targeted PET-CT imaging of peripheral angiogenesis in diabetic mice. Sci. Rep..

